# Clinical Symptoms, Imaging Features and Cyst Distribution in the Cerebrospinal Fluid Compartments in Patients with Extraparenchymal Neurocysticercosis

**DOI:** 10.1371/journal.pntd.0005115

**Published:** 2016-11-09

**Authors:** Rodrigo Bazan, Pedro Tadao Hamamoto Filho, Gustavo José Luvizutto, Hélio Rubens de Carvalho Nunes, Newton Satoru Odashima, Antônio Carlos dos Santos, Jorge Elias Júnior, Marco Antônio Zanini, Agnès Fleury, Osvaldo Massaiti Takayanagui

**Affiliations:** 1 Department of Neurology, Psychology and Psychiatry from Botucatu Medical School; Univ Estadual Paulista. Botucatu, Brazil; 2 Department of Neurosciences and Behavioral Sciences from Ribeirão Preto School of Medicine; Univ São Paulo. Ribeirão Preto, Brazil; 3 Department of Internal Medicine from Ribeirão Preto School of Medicine; Univ São Paulo. Ribeirão Preto, Brazil; 4 Institute for Biomedical Investigation; Universidad Nacional Autónoma de Mexico. Mexico City, Mexico; Universidad Nacional Autónoma de México, MEXICO

## Abstract

Extraparenchymal neurocysticercosis has an aggressive course because cysts in the cerebrospinal fluid compartments induce acute inflammatory reactions. The relationships between symptoms, imaging findings, lesion type and location remain poorly understood. In this retrospective clinical records-based study, we describe the clinical symptoms, magnetic resonance imaging features, and cyst distribution in the CSF compartments of 36 patients with extraparenchymal neurocysticercosis. Patients were recruited between 1995 and 2010 and median follow up was 38 months. During all the follow up time we found that 75% (27/36) of the patients had symptoms related to raised intracranial pressure sometime, 72.2% (26/36) cysticercotic meningitis, 61.1% (22/36) seizures, and 50.0% (18/36) headaches unrelated to intracranial pressure. Regarding lesion types, 77.8% (28/36) of patients presented with grape-like cysts, 22.2% (8/36) giant cysts, and 61.1% (22/36) contrast-enhancing lesions. Hydrocephalus occurred in 72.2% (26/36) of patients during the follow-up period. All patients had cysts in the subarachnoid space and 41.7% (15/36) had at least one cyst in some ventricle. Cysts were predominantly located in the posterior fossa (31 patients) and supratentorial basal cisterns (19 patients). The fourth ventricle was the main compromised ventricle (10 patients). Spinal cysts were more frequent than previously reported (11.1%, 4/36). Our findings are useful for both diagnosis and treatment selection in patients with neurocysticercosis.

## Author Summary

Neurocysticercosis is caused by the accidental ingestion of eggs from *Taenia solium* whose larvae lodge in the central nervous system. In this study we found that cysts within the cerebrospinal fluid leaded to high rates of raised intracranial pressure, meningitis, seizures and headache. Imaging studies such as magnetic resonance are useful for diagnosis, identification of the compromised sites of the central nervous system and, then, for treatment guidance. The pattern of lesions identified through magnetic resonance in our paper helps physicians on searching and analyzing some typical findings of extraparenchymal neurocysticercosis.

## Introduction

Neurocysticercosis is the most common parasitic disease of the central nervous system (CNS) worldwide. It is caused by the accidental ingestion of eggs of the tapeworm *Taenia solium*. After hatching from the eggs, some of the larvae lodge themselves in the CNS, where they induce inflammatory reactions causing various symptoms[1]. Although neurocysticercosis has potential for eradication, it is a neglected disease that remains endemic in Latin America, Sub-Saharan Africa, and Southeast Asia [2]. In addition, migratory flows have reintroduced the disease in Europe and the USA [3, 4], increasing public health expenditure. [5].

There are two main forms of neurocysticercosis, parenchymal and extraparenchymal. Cyst localization determines the clinical presentation of the patient. Cysts within the brain parenchyma are responsible for seizures that generally respond well to antiepileptic drugs. In such cases, the prognosis is good unless there is a large parasite burden. In contrast, extraparenchymal neurocysticercosis has a very poor course with high rates of mortality and disability [6–8]

Cysts in the cerebrospinal fluid (CSF) compartments elicit inflammatory responses that can cause vasculitis, meningitis, CSF circulation imbalance, and elevated intracranial pressure. Consequently, such cysts can cause strokes, hydrocephalus, and death [9–12]. Cysts in the subarachnoid space over the convexity of the brain develop and progress similarly to parenchymal cysts [1]. It is hypothesized that cysts located extraparenchymally may continuously absorb the CSF and may grow to large sizes, resulting in compression of adjacent structures. This phenomenon is called hydropic degeneration and is implicated in the transformation of the cellulose into the racemose type of cysticerci [13]. Generally, scolices are absent in parasites located in these regions, and the cysts show a characteristic morphology with complex proliferating membranes resembling a cluster of grapes [14, 15]. In the brain ventricles, the cysts can cause ependymitis and direct obstruction of the CSF flow, which in turn results in hydrocephalus and often requires surgical intervention [16, 17].

Computed tomography (CT) and magnetic resonance imaging (MRI) are important tools for the management of neurocysticercosis used for both diagnosis and treatment decision making. CT provides useful information on the stage of parenchymal parasite development; however, it lacks accuracy for the identification of cysts in the CSF compartments because CSF and cyst fluid show similar attenuation on X-rays. MRI enables better visualization of cysts within the subarachnoid space and ventricles [18]; particularly new MRI sequences with improved sensitivities allow earlier diagnosis [19].

Although many reports describe and review the clinical and radiological presentations of neurocysticercosis, there is a dearth of systematic studies of lesion site distribution and its correlations with symptom development. In this study, we describe the symptoms and radiological findings with special attention to cyst locations in a cohort of patients with extraparenchymal neurocysticercosis.

## Methods

### Ethics statement

This project was approved by the Research Ethics Board from the Hospital das Clínicas da Faculdade de Medicina de Ribeirão Preto (HCRP 12274/2006). All the patients gave informed consent and analyzed data were anonymized.

This is a retrospective cohort study conducted at a specialized ambulatory clinic of a third level hospital in Ribeirão Preto, Southeastern Brazil, an endemic area for neurocysticercosis. Patients were both from rural and urban areas and were attended by the Brazilian Public Health System. Rural areas are defined administratively by Brazilian municipalities, but the official calculations results are close to the Organisation for Economic Co-operation and Development one’s [20].

**Fig 1:**
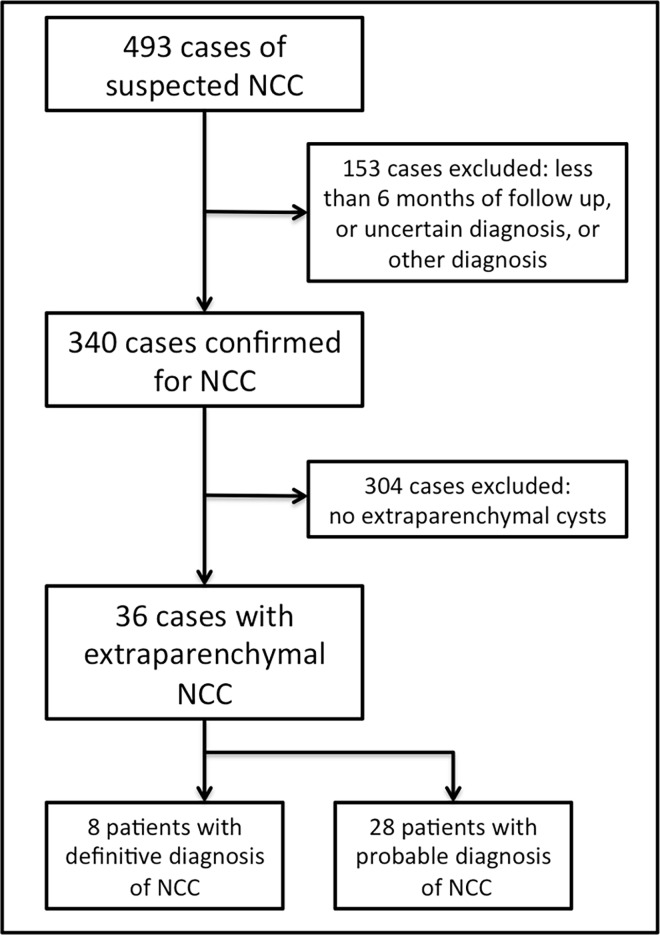
Flowchart of participants recruitment. There were included 36 patients. By the end of the follow up, eight patients had definitive diagnosis of neurocysticercosis by fulfilling the absolute criterion of histological demonstration of the parasite after surgical removal. Beside the evidence of lesion highly suggestive of neurocysticercosis on neuroimaging studies and clinical manifestations suggestive of neurocysticercosis, 21 patients had positive ELISA and 7 patients had an epidemiological criterion.

From 1995 to 2010, a total of 493 patients were evaluated for the suspicious of neurocysticercosis and 340 cases were confirmed. Other diagnosis included brain neoplasms and other CNS infections. From the 340 confirmed cases, 36 of them (10.6%) had extraparenchymal neurocysticercosis. Fig 1 summarizes the recruitment of participants. Inclusion criteria were: patients of any age, both sexes, with MRI studies showing characteristic lesions of neurocysticercosis and with a minimum follow up period of six months. Exclusion criteria were: uncertain diagnosis of neurocysticercosis, follow up period lesser than six months. We analyzed the medical records of the 36 patients diagnosed with extraparenchymal neurocysticercosis. Symptoms presented at admission and during the follow-up were grouped in four clinical syndromes: raised intracranial pressure, cysticercotic meningitis (meningeal irritation signs ie. nuchal rigidity and Brudzinski or Kernig signs), seizures and isolated headache. In all patients, at least one 1.5T MRI examination of the brain was performed with the pattern sequences of T1, T2 and FLAIR. An initial MRI was performed for diagnosis purposes and subsequent examinations were performed on the basis of clinical indications. Patients with symptoms of spinal or radicular compression also performed a spine MRI according to the clinical needs. Median follow-up period was 38 months (range 8.8 to 156 months). We used the imaging data to analyze the distribution of the cysts among the subarachnoid cisterns, brain fissures, ventricles, and spine. We also analyzed the size of the cysts, the presence of contrast-enhancement, the pattern of growth and the association with parenchymal lesions, and occurrence of hydrocephalus. Multiple cystic lesions resembling a bunch of grapes were considered “grape-like lesions”. Cysts that reached more than 50mm at the largest dimension were considered giant.

For statistical analysis, we used Fisher’s exact test and the Chi-square test for comparison of percentage between groups. SPSS (v. 15.0) and GraphPad Prism (v. 4.0) softwares were used.

## Results

### Clinical findings

The patients were aged from 21 to 65 years (40 ± 9.8 years). Twenty were male (55.5%), and about 22.0% (8/36) lived in rural areas. By the admission, all patients had a probable diagnosis, according to Del Brutto’s criteria [21]. By the end of the study, eight patients had a definitive diagnosis of neurocysticercosis after pathologic confirmation, and 28 maintained the probable diagnosis, as shown in Fig 1.

#### Initial clinical presentation

Headache was the most common initial symptom, affecting 77.8% of the patients (28/36). Nausea and vomiting affected 21 patients (58.3%), seizures 16 (44.4%) and fatigue 8 patients (22.2%). On neurological examination, fourteen patients presented no abnormality (38.9%). Papilledema was present in 17 patients (47.2%), slight motor deficits in 8 patients (22.2%), signs of meningeal irritation were observed in 7 patients (19.4%), cranial nerve deficits (VI, VIII and XII) in 5 patients (13.9%), cognitive impairment in 4 patients (11.1%) and ataxia/dysmetria in 2 patients (5.6%).

Combining symptoms and signs, we observed the following most common syndromes and diseases: raised intracranial pressure in 58.3% (21/36); seizures in 44.4% (16/36); headache (without raised intracranial pressure) in 25.0% (9/36); and cysticercotic meningitis in 19.4% (7/36) of the patients.

#### Clinical presentation during follow up

During the follow-up period the patients changed their clinical pictures and presented new symptoms and syndromes. The mean duration of time from the initial diagnosis to the onset of new syndromes was 6.0 months for meningitis, 27.3 months for seizures, 36.3 months for raised intracranial pressure, and 55.6 months for chronic headache.

In the entire observational period, the most common syndromes and diseases were raised intracranial pressure (75.0% of the cohort, 27/36), cysticercotic meningitis (72.2%, 26/36), seizures (61.1%, 22/36), and isolated headache (50.0%, 18/36). Prompt treatment was offered to patients as soon as new symptoms or complications appeared. Painful radiculopathy and sudden deficit symptoms related to stroke were also found, albeit less frequently (11.1%, 4/36; and 8.3%, 3/36 respectively). Raised intracranial pressure was more common in men than in women (90.0% vs. 56.2%, *p* = 0.049). Isolated headache was more common in women than in men (75.0% and 30.0%, respectively; *p* = 0.017). Among the patients with headaches, migraine was found in 44.4% (8/18), tension headache in 38.9% (7/18), and chronic daily headache in 16.7% (3/18) of the patients.

Twenty two patients (61.1%) were treated with anti-parasitic drugs (21 of them with Albendazole). Corticosteroids were administered to 28 patients (77.8%). Twenty four patients (66.7%) had at last one surgical procedure (the most frequent was ventriculoperitoneal shunt [VPS], in 23). Microsurgical removal of cysts was performed in seven patients (19.4%) and endoscopic surgery for cyst removal and third ventriculostomy was performed in other seven patients. Among the patients with VPS, 65.2% (15/23) presented shunt malfunction sometime, and 26.1% (6/23) presented a shunt-related infection. Table 1 summarizes the clinical findings. Four patients (11.1%; all male) died during the observational period. These four patients presented seizures sometime, had increased intracranial pressure and died after decompensation of hydrocephalus. On neuroimaging studies, all had cysts in the posterior fossa and in the basal cisterns. From the 32 remaining patients, twelve had good outcome, without permanent deficits, and twenty had unfavorable outcome, with some sequel.

**Table 1:** –Clinical findings of the cohort of patients studied from 1995 to 2010.

Demographic data	
Age at diagnosis (mean, sd)	40 years, 9.8
Sex	20 men (55.5%); 16 female (45.5%)
Habitation	28 urban (78%); 8 rural (22%)
**Initial clinical symptoms and signs**	
Headache	28 (77.8%)
Nausea/vomiting	21 (58.3%)
Seizures	16 (44.4%)
Fatigue	8 (22.2%)
Papilledema	17 (47.2%)
Motor deficits	8 (22.2%)
Meningeal irritation	7 (19.4%)
Cranial nerve deficits	5 (13.9%)
Cognitive impairment	4 (11.1%)
Ataxia/dysmetria	2 (5.6%)
**Initial clinical syndromes**	
Raised intracranial pressures	21 (58.3%)
Seizures	16 (44.4%)
Headache (w/o raised intracranial pressure)	9 (25%)
Cysticercotic meningitis	7 (19.4%)
**Clinical syndromes during all the follow-up period**	
Raised intracranial pressure	27 (75%)
Cysticercotic meningitis	26 (72.2%)
Seizures	22 (61.1%)
Headache (w/o raised intracranial pressure)	18 (50%)
**Treatment modalities**	
Anti-parasitic drugs	22 (61.1%)
Corticosteroids	28 (77.8%)
Ventriculoperitoneal shunt	23 (63.9%)
Microsurgical removal of cysts	7 (19.4%)
Endoscopic approaches	7 (19.4%)

### Imaging findings

#### Types and developmental stages of parasites

**Fig 2:**
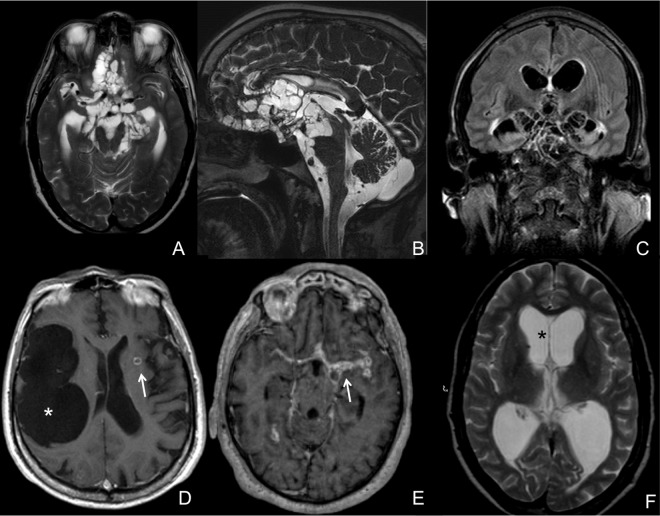
Different presentation patterns of extraparenchymal neurocysticercosis as revealed by brain MRI. Grape-like lesions in the basal cisterns are evident on T2 axial (A) and sagittal (B) views of a patient’s brain. C, An axial fluid-attenuated inversion recovery (FLAIR) coronal view of the same patient showing lesions in the Sylvian and interhemispheric fissures as well as basal cisterns. D, A giant cyst (*) in the right Sylvian fissure and a parenchymal enhancing cyst (arrow). E, A T1 axial view showing contrast enhancing in the Sylvian fissures (arrow), mainly on the left, which is associated with intense inflammatory reactions due to degenerating cysts. F. Ventricle enlargement (*) in a patient with neurocysticercosis-associated hydrocephalus.

Grape-like lesions were observed in 77.8% (28/36) of the patients (Fig 2A–2C). Eight patients (22.2%) had giant cysts (Fig 2D), and 6 (16.7%) had both grape-like and giant cysts. In 22 patients (61.1%), there was contrast enhancement near the cyst membranes (Fig 2E). Twelve patients presented a cyst mass (compression) effect; in 10 of them, the brainstem was compressed. In the other 2 patients, the compressed regions were the optic chiasm and the pituitary stalk. Associated parenchymal cysts were found in 27 patients (75%). Twenty-three patients (63.9%) had calcified lesions, 7 cysts at the vesicular stage, and 6 colloidal cysts. Four patients had both calcified and colloidal cysts, three patients had calcified and vesicular cysts, and one patient had calcified, colloidal and vesicular cysts. Twenty-six patients (72.2%) presented hydrocephalus sometime during the observational period (Fig 2F).

#### Cyst distribution in the CNS

**Fig 3:**
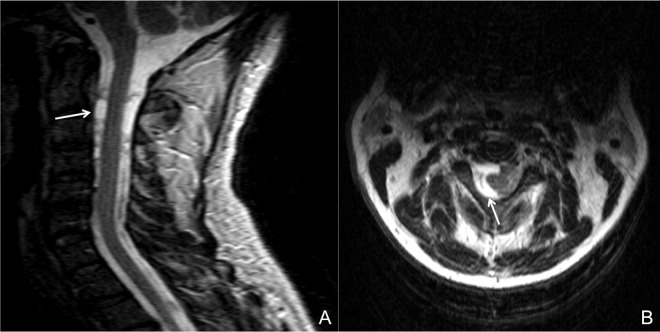
Spinal cysts. A spinal cyst (arrows) at the cervical level in a patient with extraparenchymal neurocysticercosis (A, sagittal view; B, axial view).

Regarding the distribution of cysts among the CSF compartments, all patients had cysts in the subarachnoid space. 31 patients (86.1%) had lesions in the cisterns of the posterior fossa near the brainstem. Nineteen patients (52.8%) presented cysts in the supratentorial basal cisterns (i.e., the perimesencephalic cisterns and supraselar cisterns near the optic chiasm and carotid artery). Fifteen patients (41.7%) had cysts in the Sylvian fissure (either right or left), 9 (25%) in the choroidal fissure, and 5 (13.9%) in the interhemispheric fissure. Four patients (11.1%) had spine cysts, 3 of them at the lumbosacral level and 1 at the cervical level (Fig 3). Only 1 patient presented cysts in the cortical sulci.

**Fig 4:**
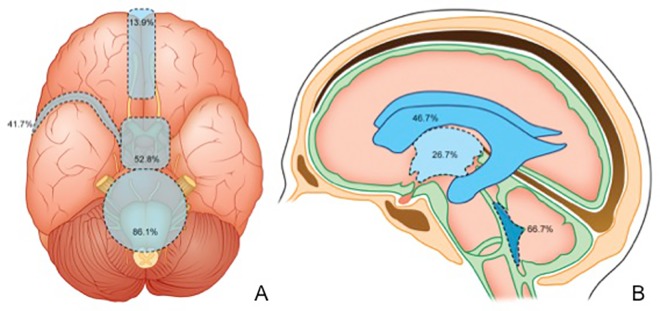
Schematic representation of the distribution of cysts in the study cohort among the CSF compartments. A, In the subarachnoid space, the posterior fossa cisterns were involved in 86.1% of patients, the supratentorial basal cisterns in 52.8%, the Sylvian fissures in 41.7%, and the interhemispheric fissure in 13.9% of patients. B, Among the patients with cysts in the ventricles, 66.7% had cysts in the fourth ventricle, 46.7% in the lateral ventricles, and 26.7% in the third ventricle. NB: percentages are higher than 100% because a single patient could have more than one compromised site.

**Fig 5:**
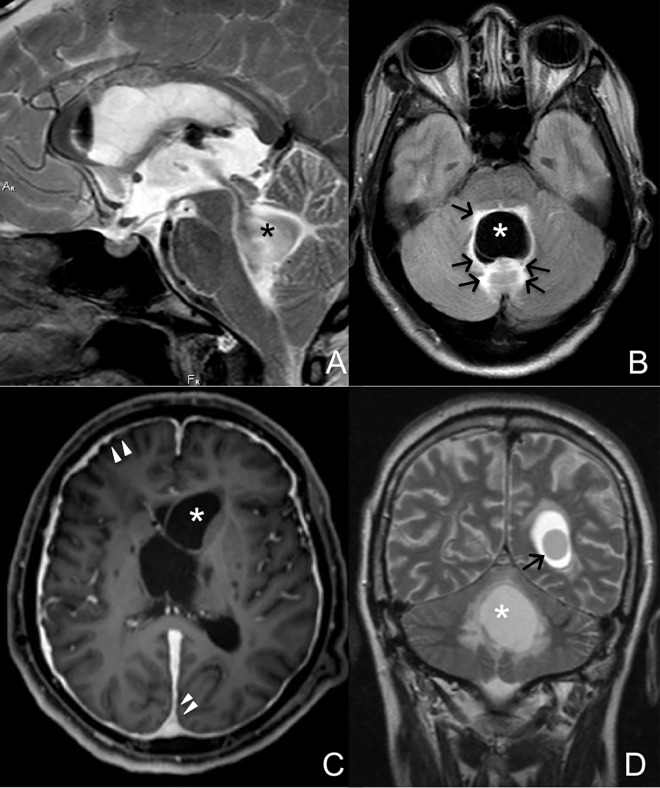
MRI images of cysts inside brain ventricles. The fourth ventricle is the most common site for ventricular neurocysticercosis. A large cyst (*) in the fourth ventricle (A) resulted in perilesional edema (arrows) in the patient’s posterior fossa (B). The lateral ventricles are also common sites of cyst location (C). Meningeal enhancement (arrowheads) is in a patient with a cyst (*) inside the left lateral ventricle. In some patients, multiple ventricles can be compromised. D, Cysts in the left lateral (arrow) and fourth (*) ventricles of a patient.

Fifteen patients (41.7%) had at least one cyst within the ventricles: the fourth ventricle contained a cyst in 10 patients, the lateral ventricles in 7 patients, and the third ventricle in 4 patients (Figs 4 and 5). Twelve patients (33.3%) had an enlarged fourth ventricle, independently of the presence of cysts within it. In 4 patients, the cyst inside the ventricles displaced adjacent structures. No patient had cysts only in the ventricles (without subarachnoid space involvement). Fig 4 summarizes the observed frequencies of cyst occurrences in the CSF compartments, including the ventricles.

### Clinico-radiological correlation

Besides sex and clinical syndromes, we also tested the correlation between the presence of ventricular cysts and sex; the presence of ventricular cysts and hydrocephalus; the occurrence of seizures and the presence of parenchymal cysts; and the presence of calcified cysts and headache.

Men have presented more ventricular lesions than women (60% vs. 18.8%, *p* = 0.019). The association between the presence of ventricular lesions and the occurrence of symptoms related to elevated intracranial pressure was close to statistical significance (*p* = 0.051). The other correlations between clinical presentation and lesion topography did not reach statistical significance (seizures and parenchymal cysts: *p* = 0.712; calcified cysts and headache: *p* = 0.298).

## Discussion

Neurocysticercosis is a pleomorphic disease. Its clinical presentation depends on the site, size and stage of cyst as well as the intensity of the inflammatory reaction generated by host-parasite interaction. A systematic review of clinical manifestations of neurocysticercosis showed that seizures and headache are the most common symptoms in patients seen in neurologic clinics [22]. Our study showed that headache was the commonest symptom on initial presentation and this is due to the fact that we focused on patients with the extraparenchymal form of the disease, which has higher rates of raised intracranial pressure.

In the brain parenchyma, seizures are the most frequent symptom, in relation with the epileptogenic properties of the parasite itself and of the local inflammatory response [23]. In our study, a high proportion of the patients presented seizures (61.1%), and despite the high frequency of parenchymal cysts, seizures did not reach statistical association with parenchymal cysts (*p* = 0.712).

On the other hand, when parasites are located extraparenchymally, it is clear that raised intracranial pressure and signs of meningitis are the most frequent symptoms. The induced inflammatory process and death of the parasites cause basal arachnoiditis, which perturbs the free circulation of CSF and caused these two types of symptoms [10]. Symptoms of raised intracranial pressure were among the initial findings in 58.3% of our patients; however, 75% had experienced these symptoms by the end of the follow-up period.

Regarding meningitis, almost 1 of 5 patients presented symptomatology while in the entire period of follow-up nearly 2/3 of the patients developed some symptom. Actually, previous reports have shown a widely variable occurrence of meningeal signs, from 44 to 80% [24–26].

There seems to be a relationship between calcified neurocysticercosis and primary headache disorders related to a dynamic remodeling process that occurs in the calcified cysts [27]. We found that 50% of our patients presented with headache unrelated to intracranial pressure changes but it was not dependent on the presence of calcified cysts (*p* = 0.298). Probably the high prevalence of migraine in the general population poses difficulty in understanding the causes of primary headache in extraparenchymal neurocysticercosis. Also, it is evident that these forms of severe NCC can produce headaches by multiple mechanisms, principally inflammatory, weakening the link between calcifications and headaches. Further research is needed to ascertain a causal link between the disease and the symptom.

The association between neurocysticercosis and cerebrovascular disease is well established [9]. Angiographic studies have demonstrated that almost one half of the patients with subarachnoid neurocysticercosis may have evidence of cerebral arteritis, mainly in the middle and posterior cerebral arteries [28]. Reduction of cerebral blood flow was also observed in patients with inflammatory pattern of CSF [29]. Near 10% of our cohort presented stroke, which is in accordance to the literature [7].

Imaging studies play a key role in diagnosing neurocysticercosis. Its clinical symptoms are variable and nonspecific, and depend on the localization of the cysts within the CNS. Though serological tests can help in diagnosis, they may not characterize the CNS involvement in cysticercosis [15]. Therefore, imaging studies are required for the most accurate diagnosis and best choice of treatment, because both depend on the activity and viability of the parasites in the CNS.

Before the advent of CT, the diagnosis of neurocysticercosis was a challenge, and the confirmation of the presence of parasites in the brain depended on risky surgical procedures [30]. CT became a useful tool for diagnosing parenchymal lesions and, to a large extent, for characterizing the development stage of the cyst. However, CT was inadequate for detecting cysts within the CSF compartments. Metrizamide CT ventriculography was an option in suspected cases of ventricular involvement; however, it was invasive and insufficient for diagnosing cysts in the fourth ventricle and basal cisterns. The higher sensitivity of MRI made it possible to detect cysts in the CSF compartments [14]. However, small cysts in early stages of development often escaped detection. Recently, high-resolution magnetic resonance cisternography sequences have improved the identification of such cysts [15, 19].

Studies have reported conflicting rates of cyst distribution in the CNS of patients with neurocysticercosis [14, 31–35]. The likely reason for the discrepant data is that most of the studies focused on general imaging findings of neurocysticercosis or specific therapeutic modalities for a given cyst localization. For example, Fleury et al. (2004) found that between the parasites located in the subarachnoid space, 61.3% of them lie within the basal cisterns and 38.7% in the cortical sulci [33]. On the other hand, pathological studies show that cortical sulci are probably the most common location of intracranial cysticerci–up to 69% in asymptomatic patients according to Sáenz et al. (2008) [36]. In contrast, we found few cysts in the cortical sulci, although the prevalence of cysts in the basal cisterns was also high in our cohort. This difference may be related to the fact that we did not include patients without extraparenchymal cysts, as long as many cortical sulci cysts may resemble cortical cysts on neuroimaging studies. Reports to date indicate that cysts in the cortical sulci behave biologically similarly to parenchymal cysts as long as they can also calcify [1]. Our findings also show predominant cyst localization in the infratentorial cisterns than in the supratentorial ones (86.1% vs. 52.8%).

Giant cysts in the subarachnoid space are found in 12.9 to 37% of patients [11, 37]. In our cohort, this proportion was 22.2%. However, the definition of a giant cyst varies. Proaño et al. (2001) used the cutoff value of 50 mm in diameter to enroll 33 patients with giant cysts out of a total of 320 patients with neurocysticercosis (10.3%) [12].

Symptomatic spinal neurocysticercosis is not very frequent–it occurs in only 0.25–5.8% of all neurocysticercosis patients. However, routine spine imaging in patients with intracranial subarachnoid neurocysticercosis revealed a much higher frequency of spinal involvement [38]. Consistent with these results, we found a relatively high rate of spinal cysts (11.1%), but our patients did had symptomatic lesions in the spine. In agreement with previous reports, the lumbar level was the most frequently affected, causing in all of our patients symptoms of painful radiculopathy.

We also found a higher rate of ventricle-associated cysts (41.7%) than most common estimates. In the series of Fleury et al. (2004), among 75 patients with extraparenchymal cysts only 18 (24%) had ventricular lesions [33]. In accordance with the literature, [32, 35, 39], we found the most cysts inside the fourth ventricle (66.6%). However, the higher rate of lateral ventricular involvement compared to that of the third ventricle differs from previous reports [40].

Hydrocephalus occurs in about 30% of patients with neurocysticercosis [6]. In patients with the extraparenchymal form of the disease, this rate rises to 64% [41]. We found an even higher rate (72.2%), probably because our patients were followed longer and, consequently, had more time to develop hydrocephalus during the follow-up period. Hydrocephalus is a major concern for patients with extraparenchymal neurocysticercosis, because ventricular-peritoneal shunts have been plagued with high rates of malfunction and risk of infection. The higher the number of surgical revisions needed, the higher the overall mortality [42, 43].

We found no statistically significant correlation between any of the symptoms and cyst topography. This may be due to the high rate of co-occurrence of cysts in different compartments. We did demonstrate a strong association between ventricular lesions and symptoms related to increased intracranial pressure that was just below the threshold of statistical significance (*p* = 0.051). In a previously described subset of patients who underwent CSF analysis, we also could not demonstrate statistically significant differences related to sex, other demographic data, or cyst location [44].

Finally, the high proportion of patients with contrast-enhancing lesions (61.1%) may be due to medical treatment, as long as we analyzed all MRI examinations during the follow-up time. Degenerating cysts induce inflammatory response that becomes evident on contrast-medium MRI sequences. It is important to be able to recognize this imaging pattern, because it poses risk for the surgical resection of cysts adjacent to vessels, nerves, and parenchymal tissue itself [45].

In the present study, we offer a systematic analysis of the distribution pattern of extraparenchymal cysts in the CSF compartments in patients with neurocysticercosis. Limitations of the present work include its retrospective nature and the limited number of patients. Its major strength is in its focus on a specific cohort of patients with the extraparenchymal form of neurocysticercosis. The presence of a scolex in a lesion is an absolute requirement in Del Brutto’s proposed criteria for a diagnosis of neurocysticercosis [21]. However, racemose cysts often lack a scolex or the scolices cannot be visualized in common neuroimaging studies. So in patients with the extraparenchymal form of the disease, it is very important to look for other criteria of the set to reach a definite diagnosis.

In our cohort of 36 patients with extraparenchymal neurocysticercosis, we found high prevalences of meningitis, seizures, headache, and symptoms related to raised intracranial pressure. MRI showed most cysts lodged in the basal cisterns, mainly in the posterior fossa. The Sylvian fissure was another common localization of cysts in general, and the most frequent site for giant cysts. Spinal cysts may be more common than previously believed. Intraventricular cysts are relatively frequent, especially in the fourth ventricle. Hydrocephalus and contrast-enhancing lesions are common features in extraparenchymal neurocysticercosis. The limited number of patients in the study precludes far-reaching generalizations. Nevertheless, our findings will be of interest for clinicians, epidemiologists and radiologists.

## Supporting Information

S1 Checklist (DOC)

S1 Database (XLS)
